# Supplementary material for: Are carbon emissions trading and green financial instruments synergistic? -Comprehensive quantitative research based on content analysis
Source: PLoS One. 2024 Mar 7;19(3):e0298601. doi: 10.1371/journal.pone.0298601 (PMC10919601; doi:10.1371/journal.pone.0298601)
Supplement: S1 Data — (ZIP) [file pone.0298601.s001.zip › Data/地级市Z_F工作报告.zip!/╜¡╦╒/│ú╓▌╩╨/└┤╫╘╣1⁄2╓┌║┼í╛┬φ┐╦╩2╛▌═°í┐_2017.docx]

各位代表：

现在，我代表常州市人民政府向大会报告工作，请予审议，并请市政协各位委员和其他列席人员提出意见。

一、本届政府工作回顾大清相国

2012年6月以来，本届政府在中共常州市委的正确领导下，在市人大、市政协的监督支持下，紧紧依靠全市人民，主动适应经济发展新常态，解放思想，开拓创新，真抓实干，奋力拼搏，胜利完成了市十五届人大历次会议确定的各项任务，全市经济社会发展迈上了一个新台阶。获批国家历史文化名城，创成国家生态市、国家森林城市，摘得中国人居环境综合奖。

（一）全力以赴调结构、促转型，综合实力不断提升。大力推进“三位一体”工业经济转型升级战略和现代服务业三年行动计划，产业结构实现“三二一”的标志性转变，全市经济发展的质量和效益显著提高。主要经济指标稳健增长。全市地区生产总值连跨两个千亿元台阶，2016年达5773.9亿元，按可比价年均增长10%，人均地区生产总值达1.85万美元，达到中等发达国家水平，稳居城市综合竞争力全国前30强。一般公共预算收入由2011年的350.9亿元增加到2016年的480.3亿元，年均增长6.5%。规模以上工业总产值跨入万亿级城市行列，2016年达1.23万亿元，年均增长9.7%。社会消费品零售总额年均增长12.5%。“三位一体”工业发展成效显著。2016年，十大产业链产值占规模以上工业比重达34.3%，汽车及零部件、碳材料、通用航空等产业链实现重大突破，重工业比重由2011年的81.5%降至2016年的76.9%，高新技术产业产值占比达44.5%，涌现出200多家国内外行业“隐形冠军”，省级特色产业集群达9个。现代服务业加快发展。2016年服务业增加值占比达50.9%，较2011年提升8.5个百分点。金融、物流、电子商务、旅游、科技服务业等保持较快增长，凌家塘批发市场、夏溪花木市场、邹区灯具城年交易额位居全国前列，“华贸通”、国家智慧旅游公共平台等一批互联网平台相继打造，东方盐湖城、迪诺水镇等一批旅游新项目建成投运，天目湖旅游度假区成为国家级旅游度假区。现代农业持续推进。农业现代化工程水平指数位居全省第二，农业现代化建设水平列全省第三，被认定为国家现代农业示范区，全市高标准农田面积、高效设施农业面积、高效设施渔业面积比重分别超过61%、21%、36%。成功举办第八届中国花卉博览会。

（二）多措并举抓创新、促改革，发展活力明显增强。坚持创新发展，全面深化改革，加快对外开放，大力塑造更多依靠创新驱动的引领型发展。重大项目有效助推经济转型。围绕建设全国一流的智能制造名城，大力引进成长性好、技术先进、带动力强的大项目、好项目。连续开展重大项目主题年活动，共引进100亿元或10亿美元以上项目9个，50亿元或5亿美元以上项目18个，其中东风、北汽、众泰等整车项目和中航锂电池等关键配套项目加速形成汽车全产业链，百度大数据产业园、中兴能源云计算、北京通航、同方威视等特色项目促进了新兴产业加快发展。创新驱动效应更加明显。大力推进国家创新型城市和苏南国家自主创新示范区建设，常州科教城连续位列中国最佳创业园区前列，武进高新区升级为国家级高新区，西太湖科技产业园、江苏中关村科技产业园获批筹建省级高新区，常州科技街启动建设，“一核两区多园”的区域创新格局基本形成。加快集聚优质创新资源，持续深化产学研合作“常州模式”，深入实施“龙城英才计划”，入列省产业技术研究院的公共研发机构数量居全省前列，引进落户领军型创新创业人才达1076名，每万名劳动者中高技能人才数居全省第一。加大知识产权保护力度，获得国家知识产权示范城市称号。改革开放不断深化。积极实施产城融合综合改革，19个市级试点改革区域有序开展。实施部分行政区划调整，金坛撤市设区，整合设立常州经开区，新北、天宁、钟楼发展空间扩大，全市生产力布局进一步优化。苏皖合作示范区纳入苏皖两省战略合作协议。商事制度改革、投融资体制改革、价格机制改革等重要领域改革深入推进，发展环境进一步优化。加快企业上市步伐，全市上市企业达43家、新三板挂牌企业106家。农村改革稳步推进，农地承包经营权确权登记颁证、农村集体产权制度改革、武进农村土地制度改革试点等工作进展顺利。大力推进“五个国际化”建设，五年实际到账外资超过150亿美元，外贸进出口总额超过1420亿美元，世界500强入驻企业达65家、投资项目104个。中以、中德等创新园区成为国家间合作项目，常州机场完成4E级改造并实现一类口岸开放，常州、武进出口加工区获批综保区。

（三）持之以恒优环境、提品质，城乡建设展现新貌。持续推进新型城镇化，城市建设实现由“筑形态”向“优品质”转变，新城新区功能日益完善，新龙国际商务城、钟楼新城、凤凰新城等城市新板块形态初显。综合交通体系不断完善，常州港建成首个10万吨级码头，西绕城高速公路、京沪高铁常州北站、青洋路高架北延等一批重大基础设施建成投运，完成丽华南路、晋陵南路、新堂北路等一批城市道路的改造提升，城市轨道交通建设加快推进。城市防洪包围圈基本形成，新孟河、新沟河延伸拓浚等一批重点水利工程进展顺利。完成两轮国家农田水利重点县工程建设。生态文明建设深入推进，连续4年在全省考核中名列前茅，丁塘河湿地公园、高铁生态公园、新龙生态林一期等一批生态项目建成开放，天目湖国家森林公园正式授牌，溧阳长荡湖湿地公园和天目湖湿地公园获准建设国家级湿地公园，森林城市和生态绿城建设取得显著成效。大气和水环境质量持续改善，2016年市区PM2.5平均浓度较2015年下降10.49%，全市空气质量优良率达70.5%，饮用水水源地水质稳定达标，太湖流域重点考核断面达标率达60%，达到国家、省考核标准要求。城市环境综合整治扎实推进，基本实现主次干道“路畅、街美、景优”，城市长效综合管理拓展至所有乡镇，成为首批“江苏省优秀管理城市”。城乡一体发展取得积极进展，持续推进“四个西进”和区县挂钩合作，建成常溧高速公路、常金快速通道等一批重大基础设施，中心镇实力进一步增强。全市村庄环境整治达标工程全面完成，建成8个省级美丽乡村示范村。土地节约集约利用水平显著提升，成为全国国土资源节约集约模范市。

（四）加大力度惠民生、促和谐，社会事业持续发展。始终坚持民生优先，持续实施民生幸福工程，民生类支出占财政总支出的比例超过75%，人民群众获得感、幸福感显著增强。人民生活持续改善。大力实施居民收入倍增计划，2016年城乡居民人均可支配收入分别达46058元和23780元，是2011年的1.57倍和1.64倍。积极推进就业创业，五年累计新增城镇就业64.5万人，城镇登记失业率控制在4%以内，成为全国创业先进市。强化社会保障和社会救助，社会保障水平稳步提高，三大保险覆盖面稳定在98%以上，在全省率先建立全市统一的城乡居民基本医疗保险制度。着力抓好棚改工作，城镇中低收入住房困难家庭住房保障应保尽保。茅山老区帮扶工作深入开展，老区面貌持续改善。社会事业优质发展。教育现代化扎实推进，五年新建和改扩建中小学、幼儿园140所，所有辖市、区均成为全国义务教育发展基本均衡县（市、区），基础教育质量位居全省前列，在常高校稳步发展，科教城获批国家高职教育综合改革试验区。卫生计生服务能力不断提高，基本建成覆盖城乡的医疗服务体系和15分钟健康服务圈，公立医院全面实施医药价格综合改革。养老服务体系基本建立，全市社区居家养老服务中心（站）实现城乡全覆盖，医养融合向纵深发展，每千名老人拥有养老床位数48张。体育事业蓬勃发展，成功创建省公共体育服务体系示范区，培养输送的运动员在奥运会等重大赛事中成绩取得历史性突破。文化名城建设深入开展，成功创建省级公共文化服务体系示范区，四级文化设施网络基本实现全覆盖，全国文明城市建设水平保持全国、全省前列，“道德讲堂”全国推广，“文化100”等文化惠民活动每年惠及市民均超百万，中国大运河（常州城区段）被列入世界遗产名录，青果巷历史文化街区、文化广场等建设项目进展顺利。社会治理不断创新。平安常州、法治常州建设深入开展，连续实施两轮治安防控体系建设三年规划，荣获全国社会治安综合治理最高奖“长安杯”。社区自治水平稳步提高，减负增效成效明显，全市城乡和谐社区建设水平位列全省前茅，“政社互动”覆盖所有镇、街道。每年办好十件民生实事，解决了一批关系群众切身利益的问题，得到市民的认可和好评。

（五）矢志不渝转职能、改作风，政府自身建设得到加强。按照“5416”基本框架推进行政审批制度改革，先后取消下放行政审批事项181项，取消所有非行政许可审批事项，市级行政审批事项精简45.8%。整合设立市级统一的公共资源交易平台，推进全市12345政府公共服务平台整合，建立全市不动产统一登记交易制度。建设项目“五联合一简化”联审联办做法得到国务院主要领导充分肯定。全面完成本轮地方政府机构改革，有序推进事业单位分类改革，全市机关事业单位在编在职人员实现负增长。扎实开展政府系统党的群众路线教育实践活动、“三严三实”专题教育和“两学一做”学习教育，严格执行中央八项规定精神和省、市有关规定。严格落实党风廉政建设责任制，持之以恒正风肃纪，坚定不移惩治腐败，政府系统反腐倡廉工作取得实效。加强机关作风建设，完善重大项目、重点工作推进机制，基本建立覆盖全市、导向明确、规范管用的“六位一体”绩效管理和综合考核体系，形成“层层抓落实，责任全覆盖”的工作格局。全面深化政务公开，建立完善行政权力网上公开透明运行系统，市级部门预决算和“三公”经费预算全部公开，“常州政企通”等网络平台开通运行。积极推进依法行政，全面建立政府法律顾问制度，自觉接受市人大、政协及各民主党派的监督，五年共办理人大代表建议、政协提案2349件，妥善解决了一批经济社会发展方面的问题。

过去一年，市政府按照市十五届人大六次会议确定的各项目标任务，坚持稳中求进总基调，积极践行新发展理念，不断创新举措，狠抓工作落实，全市经济社会实现了平稳健康发展。全年地区生产总值增长8.5%；一般公共预算收入增长3%；固定资产投资增长6.5%；社会消费品零售总额增长10.7%；城乡居民人均可支配收入增长8.6%；实际到账外资25亿美元；实现进出口总额1820亿元，增长4.6%，其中出口1375亿元，增长4.2%。重点抓了以下五个方面工作：一是持续推进重大项目建设。深入开展“重大项目推进年”活动，引进时代新能源锂电池长三角基地、瑞声手机3D玻璃等4个总投资超100亿元或10亿美元项目，车和家智能汽车、恒益大通芯片载带、北汽集团常州产业基地等7个总投资超50亿元或5亿美元项目。东风汽车AX5、北汽通航P-750飞机、天域航空无人机等一批项目实现整机整车下线。二是着力推动转型创新。获批共建“中国制造2025”苏南城市群试点示范，入选省大中型制造装备升级项目数居全省第一。十大产业链产值达4213亿元，增长9.5%。“互联网+”、电子商务发展迅速，电商服务业业务收入增长超过40%。旅游业持续增长，接待国内外游客和旅游总收入分别达6004.2万人次、833.6亿元，分别增长10.1%、14%。全市高新技术企业总数达1231家，预计全社会研发投入占地区生产总值的比重达2.69%，发明专利申请量、授权量分别增长25%、30%。武进获批国家首批“双创”示范基地，常州科教城获准建设全国首个国家机器人及智能硬件知识产权保护中心，戚研所捧回我国工业最高荣誉“中国工业大奖”，中简科技成为我国高性能碳纤维产业的领军企业。三是深入推进改革开放。大力推进供给侧结构性改革，全面完成省下达的钢铁、水泥去产能任务，市区商品住房可售余量去化周期缩减到6个月以内，实体经济杠杆水平有所降低，降低企业成本150亿元，棚户区改造、生态环保等补短板工程取得积极进展。加快金融创新，成功设立注册资本均为百亿元的中德产业发展基金、中以创新发展基金，金融商务区建设顺利推进。大力实施经济国际化战略，新增协议外资超3000万美元以上项目43个，中以常州创新园共建计划正式发布，苏澳合作园区落地我市，中德创新园入选“中欧城镇化伙伴关系示范区”，西太湖科技产业园获批设立“海峡两岸（常州）健康产业合作区”。四是加大城乡建设和生态环境改善力度。完成常州市空间发展战略规划编制。122省道常州东段等道路建成通车，340省道常州东段、地铁1号线一期等在建工程进展顺利。成功申报省海绵城市建设试点城市。积极应对、成功抗击夏季特大洪涝灾害。生态绿城建设持续推进，实现增核14000亩、扩绿4000亩、连网200公里以上，全市林木覆盖率达25.9%，建成区绿化覆盖率达43.1%，人均公园绿地达14.5平方米。单位GDP能耗下降超额完成省定任务。五是强化民生保障。健全就业促进机制，城镇新增就业13万人，扶持创业1.3万人。城乡居民医保制度整合全面完成。积极推进教育优质均衡发展，常州五中等7所学校建成投用，新增省、市优质幼儿园34所，常州工学院新校区投入使用，高考全市本二以上达线率达80.56%，比上年提高3.1个百分点。深化公立医院综合改革，以三级医院为主体的区域性医联体实现主城区全覆盖，分级诊疗、家庭医生签约服务、智慧健康建设加快推进。关爱失独家庭，全面二孩政策稳妥实施。强化社会治理创新，安全生产、食品药品安全形势总体平稳，社会保持和谐稳定。

与此同时，粮食供销、外事侨台、审计统计、工商物价、质监食药监、国资国企、海关国检、人武人防、双拥优抚、边防海事、档案方志、防灾减灾、应急管理、民族宗教、老龄、妇女儿童、残疾人和关心下一代等工作都取得了新成绩。

各位代表！本届政府五年来的工作，不仅为未来发展奠定了更加坚实的基础，也积累了宝贵的经验。

一是必须坚定不移践行新发展理念，提升常州发展实力。抢抓国家、省重大战略实施的机遇，努力在创新、协调、绿色、开放、共享发展上深思善谋、积极探索，加快构建有利于常州发展的体制机制，不断拓展发展新空间、积蓄发展新能量、赢取发展新优势。

二是必须坚定不移推进重大项目，增强经济发展后劲。围绕提升产业质态，持之以恒推进带动能力强、产业层次高、产出效益好的重大项目建设，不断夯实产业基础、扩大有效投入，为经济发展带来更多优质增量，推进经济结构优化和现代产业体系建设。

三是必须坚定不移突出工作重点，打造常州发展特色。按照“一城一中心一区”的发展定位，积极探索符合常州实际、彰显常州特色的工作思路，明确以“三位一体”战略提升工业经济，以区划调整加快产城融合综合改革，以生态绿城促进生态文明建设，以“三优三安两提升”为重点打造民生品牌，全面推进经济社会科学发展、跨越发展。

四是必须坚定不移增进民生福祉，建设幸福美好家园。坚持把人民对美好生活的向往作为奋斗目标，持续加大对保障和改善民生的投入，多渠道增加居民收入，加快构建公共服务体系，着力优化人居环境，促进社会安定有序，人民安居乐业，切实增强人民群众的获得感和满意度。

各位代表！近五年发展取得的成就来之不易，这是全市上下在省委、省政府和市委的正确领导下，团结拼搏、攻坚克难、开拓创新的结果。在此，我代表市人民政府，向辛勤工作在各个领域的全市人民，向市人大代表、政协委员，向市各民主党派、工商联、各人民团体、各界人士和离退休老同志，向驻常人民解放军、武警官兵、公安政法干警和部省驻常单位，向所有关心、支持、参与常州建设和发展的中外投资者、科技工作者、务工人员和海内外朋友们，致以衷心的感谢和崇高的敬意!

在肯定成绩的同时，我们也清醒地看到工作中存在的问题和不足。主要表现在：产业层次依然不高，自主创新能力还不强，经济发展的质量和效益有待提升；城市品质仍需提高，生态文明建设任重道远；医疗、养老、交通等公共服务与人民群众的期望还有一定差距；一些政府工作人员的改革意识、服务意识、担当意识还不强等。对此，我们一定高度重视，切实采取措施，认真改进和解决。二、新一届政府工作思路和2017年主要工作

今后五年，是高水平全面建成小康社会、积极探索开启基本实现现代化建设新征程的关键期。新一届政府肩负着光荣而艰巨的使命，我们将以创新实干的实际行动，努力交出一份无愧于时代、让常州人民满意的发展业绩。新一届政府主要工作目标是：

——在“经济强”上增创新优势。围绕建设具有核心竞争力的经济强市，以提升经济发展质量和效益为中心，加大转型升级力度，持之以恒推进重大项目建设，以供给侧结构性改革、产城融合综合改革为主抓手统筹推进各项改革，以“五个国际化”为重点全面融入国家开放战略，以苏南国家自主创新示范区为支撑建设长三角特色鲜明的产业技术创新中心，以区域协调发展为导向提升整体能级，充分激活发展动能，构筑更强产业支撑，促进经济保持中高速增长，产业迈向中高端水平，加快构建现代产业体系。到2021年，地区生产总值超过8000亿元，培育2个两千亿级、5个千亿级、3个五百亿级制造业优势产业，打响全国一流的智能制造名城品牌。

——在“百姓富”上实现新提升。顺应广大人民群众过上美好生活的新期待，大力培植“常州幸福树”。努力培育富民经济，大力发展区域优势产业、特色产业，通过就业增收、创业致富、精准扶贫等多途径增加居民收入，让市民享有更优质的教育、更好的医疗服务、更周到的养老服务、更安全的食品药品、更普惠的文体服务、更宜人的居住环境，进一步完善社会保障体系、社会救助体系，持续打造以“三优三安两提升”为重点的民生品牌，不断增强人民群众的获得感和幸福感。到2021年，城乡居民人均可支配收入分别达65000元和35000元。

——在“环境美”上彰显新内涵。围绕打造国内领先的产城融合示范区，统筹推进城市升级计划，充分展现协调之美、品质之美、生态之美、特色之美，基本建成具有突出竞争力和影响力的区域中心城市。借力沿江城市集群发展、锡常泰跨江融合等提升城市地位，建设江苏中轴崛起的桥头堡和具有跨江联动效应的战略性中心城市。聚焦产城融合，优化城市品质，常州中心城区加快建设面向国际的现代化城区，金坛区全面融入主城区发展格局，溧阳市加快崛起为宁杭生态经济发展带副中心城市，打造独具优势的特色小镇、美丽乡村。深入推进生态文明建设，持续开展生态环境综合治理，打响生态绿城品牌，精致建设花园城市，建成国家生态园林城市、国家生态文明示范市，形成绿色发展的鲜明优势。

——在“社会文明程度高”上取得新进展。以社会主义核心价值观为引领，丰富城市内涵，创新社会治理，不断提升文明新高度和文化软实力。深化全国文明城市建设，构筑思想文化建设高地、道德风尚建设高地。充分彰显城市文化特色，深化国家历史文化名城保护工作，大力发展文化产业，繁荣艺术精品创作，强化地方标识系统和城市设计，形成独具魅力的城市文化气质。全面推进依法治市，不断提升法治常州建设水平。加强和创新社会治理，持续深化“政社互动”，完善社会矛盾纠纷多元化解机制，建设平安常州，促进社会和谐发展。

当前，世界经济深度调整曲折复苏，新一轮科技革命与产业变革正孕育兴起，我国经济仍处于重要战略机遇期，经济发展新常态为我们“弯道超越”创造了新的空间。我们将准确把握战略机遇期内涵的深刻变化，坚定发展信心，找准突破路径，不断开辟常州发展新境界。今年政府工作总体思路是：全面贯彻党的十八大和十八届三中、四中、五中、六中全会精神，认真落实省委、省政府和市委的决策部署，紧紧围绕“五位一体”总体布局和“四个全面”战略布局，坚持稳中求进工作总基调，牢固树立和贯彻落实新发展理念，适应把握引领经济发展新常态，坚持以提高经济发展质量和效益为中心，以供给侧结构性改革为主线，以创新为基、以富民为本，扎实开展“重大项目提升年”活动，全力打造“一城一中心一区”，全面做好稳增长、促改革、调结构、惠民生、优生态、防风险各项工作，促进经济社会平稳健康发展和社会和谐稳定，为高水平全面建成小康社会、建设“强富美高”新常州打下坚实基础。

今年经济社会发展的主要预期目标是：地区生产总值增长8%左右；一般公共预算收入增速高于全省平均水平；固定资产投资增长8%；社会消费品零售总额增长10%左右；实际到账外资和外贸进出口总额有所增长；城乡居民收入增长与经济增长同步；居民消费价格总水平涨幅控制在3%；城镇登记失业率控制在4%以内；全面完成省下达的节能减排目标任务。

为实现上述目标，我们将重点抓好六个方面工作：

（一）持续推进重大项目

1.促进重大项目建设提速。组织实施好160个省、市重点项目，切实发挥投资对稳增长、调结构的关键作用。把重大项目建设作为重中之重，加大推进力度，加快建设速度，进一步提高新建项目开工率、续建项目竣工率，加快时代新能源锂电池长三角基地、车和家新能源汽车等项目建设，推动中航锂电、今创轨道交通装备等项目投产达效，形成新的经济增长点。

2.提高重大项目招引质量。强化全要素招商理念，大力推进“引资、引技、引智”联动招商，以项目招引带动核心技术、先进管理、高级人才等先进要素集聚。根据高新技术产业和现代服务业发展趋势，主动对接各类央企、知名外企、大型民企，重点引进新兴产业的高技术项目、传统产业的高端产品项目、跨国公司地区总部项目、现代服务业新兴项目，努力取得智能装备制造、碳材料、金融服务、健康服务、大数据等领域重大项目引进新突破。建立科学的重大项目评估机制，引导项目招引从注重规模转向效益优先，提高项目招引质效。

3.优化重大项目投资环境。充分发挥中以创新发展基金、中德产业发展基金等产业基金的作用，灵活运用股权投资、资本运作、兼并重组等市场手段，撬动重大项目招引和建设。深化投贷结合、债贷结合等融资模式，集聚各类资金支持重大项目建设。积极创新项目推进机制，强化项目建设要素保障，完善行政审批绿色通道，加快储备项目转化进程，加强督查考核，提高重大项目建设成效。

（二）突破提升产业能级

1.建设智能制造名城。坚持工业立市、制造强市、质量强市，围绕建设全国一流的智能制造名城，实施智能制造、绿色制造、品质常州等行动计划，全力推进“中国制造2025”苏南城市群试点示范建设。深化“三位一体”工业转型升级战略，加快壮大轨道交通、汽车及零部件、太阳能光伏、智能数控和机器人等十大产业链，引领制造业向中高端迈进。聚焦发展智能装备制造和石墨烯产业，加快北自所国家级智能制造系统集成体验验证平台建设，争创国家智能装备产业集聚区，精心筹办全球石墨烯大会，大力建设世界知名的“东方碳谷”。加快传统优势产业转型升级，推进冶金、建材、化工、纺织服装等转向新兴产业和产业链中高端环节，着力提升产业效益。

2.创新发展现代服务业。促进现代服务业向专业化、高端化、国际化发展，重点发展金融服务、商务服务、科技服务、电子商务等产业，加快金融商务区、互联网产业园、大数据产业园、检验检测认证产业园、西太湖影视基地等特色集聚区建设，加大龙头企业培育力度，努力实现服务业总量扩大、结构优化、业态创新。以旅游、文化、体育、健康、养老、教育培训等幸福产业引领新消费，加快构建全域旅游发展新格局，壮大影视传媒、动漫游戏、数字内容等文化创意产业，加大优质产品和服务供给，不断释放潜在消费需求。深入推进放心消费创建，营造便利放心消费环境。

3.提高现代农业发展水平。建设更高水平的国家现代农业示范区，确保农业现代化建设水平走在全省全国前列。落实农业“1185”布局规划，新建高标准农田4万亩，新发展高效设施农业（渔业）1.65万亩，提升农业综合生产能力。积极培育家庭农场、专业大户、农民专业合作社、农业产业化龙头企业等新型农业经营主体，加快培育新型职业农民，推进现代农业产业园区建设。深化农业供给侧结构性改革，着力发展新产业、新业态，大力推进农业产业结构优化提升，努力实现“一二三”产业的融合发展。加大绿色优质农产品供给，启动创建国家农产品质量安全市，建设具有区域特色的休闲农业示范区，打造品质、品位、品牌兼具的“精品农业”。

4.大力振兴实体经济。实施新一轮民营经济转型升级三年行动计划，进一步创优实体经济发展环境，引导民营企业精准加力有效投资。鼓励企业发扬“工匠精神”，加大品牌培育、推广和保护力度，提高产品质量和市场竞争力，培育更多“百年老店”。推动更多的企业股改上市挂牌，借助资本市场实现裂变发展，做大上市企业集群。以“四新经济”推动实体经济发展，增强企业发展新动能。培育发展智造经济、数字经济、分享经济、绿色经济等新业态，大力发展人工智能、印刷电子、传感器等新产业。扎实推进“互联网＋”，加快国家智慧旅游平台等互联网平台经济发展。推进产业跨界融合，积极发展服务型制造，促进三次产业及产业内部细分门类联动发展。

（三）打造更强创新引擎

1.加快创新型园区建设。优化苏南国家自主创新示范区布局，扎实推进“一核两区多园”创新型园区建设，加快常州科教城东扩步伐，推动“科创走廊”和机器人及智能硬件知识产权保护中心建设，打造常州科技街等一批科创街区。完善创新型园区服务功能，建设公共技术服务平台、人才服务中心、科技金融中心和产学研合作中心。围绕建设国家知识产权示范城市，推进知识产权示范园区、知识产权密集型产业和企业培育、高价值专利培育示范中心建设，全年发明专利申请超过12000件、授权超过1700件。

2.加速创新型企业培育。完善以企业为主体、市场为导向、政产学研用资介相结合的技术创新体系，新建100家市级以上重点企业研发机构，鼓励以产业链为基础、按创新链组建产业研发创新联盟，着力增强产业创新能力。高水平建设江苏中科智能院、南大常州院等骨干公共研发机构和20多家产业协同创新平台，争取智能装备、碳材料、光伏等产业技术创新中心列入国家级序列，构建“国家级、省级、市级”梯队型创新平台。大力推进“十百千”创新型企业壮大工程，培育一批创新能力强、市场影响力大的创新型领军企业和成长速度快、发展前景好的科技“瞪羚”企业。

3.加强创新型人才集聚。把人才战略作为引领未来发展的主导战略，深入实施“龙城英才计划”，对接企业需求和产业发展方向，精准集聚精英人才，着力建设创新创业高地。瞄准十大产业链建设和现代服务业领域，大力引进世界水平的科学家、领军型创新创业人才，大力培育企业家人才、高技能人才。优化人才生态体系，建设一批国家级、省级孵化器和众创空间，加快建设武进“双创”示范基地和常州科教城国家级科技服务示范区，大力发展科技服务和科技金融，构建完善的创新创业服务体系，加强生活保障服务，提供优越的创新创业环境。

（四）更高层次推进改革开放

1.全面深化各项改革。加快供给侧结构性改革，全面落实已部署的改革任务，根据新情况新问题完善政策措施，深入推进“三去一降一补”五大任务取得新的实质性进展，着力提高供给体系质量和效率。深入推进产城融合综合改革，加快市级产城融合示范区建设，全力支持武进区国家级产城融合示范区建设，尽快形成产城融合发展的典型示范。充分发挥国家、省改革试点的作用，推进武进农村土地制度“三项改革”试点等取得新成效。坚持和完善农村基本经营制度，深化农村集体产权制度改革。加快国有企业、投融资等经济体制改革，充分释放市场活力。

2.建设高端开放平台。全面融入国家重大开放战略，着力建设更高层次、更富成效的开发开放载体。促进开发园区提档升级，推动常州、金坛、溧阳经开区争创国家级经济开发区。加强与世界创新型国家和地区的合作，加快建设中以常州创新园、中德创新园、苏澳合作园区，积极推进中芬绿色科技产业园、中荷航空产业园发展，着力打造国际合作园区特色和优势。深化跨省区域联动发展，大力支持苏皖合作示范区建设。

3.提升开放型经济引领水平。加快培育新兴产业出口基地和品牌，促进外贸集聚集约发展。加快进出口产品向“优质优价”、“优进优出”转型，支持成套设备“出海”，鼓励先进设备和关键零部件进口，提升高新技术产品进出口比重。推进常州、武进两个综保区开展跨境贸易电子商务试点，加快“华贸通”、“一达通”、“装备通”等外贸服务平台建设，发挥进境商品指定口岸作用，有效助推外贸发展。支持工程机械、轨道交通、光伏等优势产能“走出去”，建立境外生产加工基地。支持有条件的企业开展国际并购，实现产业链、价值链的全球布局。

（五）加快建设美丽常州

1.推进基础设施建设。加快提升区域交通枢纽地位，开工建设常宜、溧高、苏锡常南部通道等高速公路和沿江城际铁路，推进233国道金坛段等道路建设，建成340省道常州东段、长虹西路快速化改造等工程。常州机场建成东航基地项目，开通更多国际航班航线。加快建设常州港亿吨大港，推进德胜河“六改三”航道整治，开展常泰过江通道前期工作。进一步完善城市路网，建成飞龙西路西延、大仓路等道路，实施新机场路快速化改造、新堂北路东延、星港西路西延等工程，有序推进地铁一、二号线建设。深入开展海绵城市建设，有序建设城市地下综合管廊，继续实施魏村水厂自来水深度处理改造及天然气利用三期工程，推进“宽带常州”、“无线城市”工程，健全城市环卫设施。优化区域防洪除涝体系，实施主城区易淹易涝地区排水设施改造，基本完成新沟河工程建设，切实加快新孟河工程进度。积极创建省公交优先发展示范市，加快完善城乡公交网络，不断提升公共交通出行分担率。

2.统筹推动城乡发展。加快新一轮城市总体规划修编，推动空间、产业、土地利用、环境保护等多规融合，以高质量的规划引领高品质的城乡发展。中心城区围绕凸显文化特色，集聚发展高端服务，合理推进城市更新，促进现代商贸与文化旅游融合发展。高品质建设新龙国际商务城、西太湖滨湖新城等新片区，推进金坛常金一体发展、溧阳加速崛起，打造各具特色的产城融合发展示范区。优化镇村体系规划，加快中心镇发展步伐，推动郑陆、奔牛、邹区与主城区一体发展，因地制宜建设美丽乡村示范村。完善城市长效管理机制，推进“931”城市环境综合整治接续工程，实施城乡管理一体化考评，切实优化城乡环境。

3.深入建设生态文明。全面落实主体功能区规划，严守耕地保护、生态保护和城镇开发边界“三条红线”，积极建设国家循环经济示范市、国家低碳试点城市和国家绿色建筑示范城市，不断提升绿色发展水平。实施碧水蓝天净土工程，以“两减六治三提升”为重点，打好大气、水、土壤污染治理攻坚战，有效改善生态环境。深入实施国家森林城市建设三年行动计划，完成造林3.2万亩，进一步提升森林建设水平。加大生态绿城建设力度，推进生态绿道、健康绿城、花园城市建设等六大工程，建设新运河风光带，建成皇粮浜湿地公园、三江口公园等项目，建设30个社区体育公园，进一步优化人居环境。

（六）着力提高共享发展水平

1.持续改善人民生活。深入推进居民收入倍增计划，拓宽增收渠道，千方百计充实百姓“钱袋子”。坚持以创业带动就业，新增城镇就业8.5万人，扶持创业6000人。不断提高社会保障水平，建立完善社会大救助体系，推进主城区社会保障一体化。新开工各类棚户区改造19200套（户），基本建成14900套（户），推进公共租赁住房货币化，实施新就业大学生住房保障，确保城镇中低收入住房困难家庭应保尽保。着力增强居民住房消费能力，全年实现住房公积金扩面8万人，发放住房公积金政策性低息贷款60亿元。完善养老服务体系，加快建设城乡标准化社区居家养老服务中心，推进医养深度融合，提升养老机构综合服务功能，积极发展养老特色园区。深入实施富民强村工程，以建档立卡农村低收入人口、茅山老区为重点，实施精准扶贫、精准脱贫。

2.优化公共服务供给。推进教育现代化建设，实施田家炳高中、北郊初中钟楼分校等81个教育建设重点项目，加大学前教育支持力度，加快义务教育优质均衡发展，提升职业教育发展水平。支持在常高校加快发展，积极筹建常州大学医学院。提升国家历史文化名城品质，建设大运河文化走廊，优化青果巷历史文化街区文态、业态，加强古镇古村和名人故居的保护利用。完善公共文化服务体系，加快文化广场建设进度，建设300个基层综合性文化服务中心，开展好“文化100”等文化惠民活动。大力建设“健康常州”，加大公立医院投入，医疗、医保、医药联动推动公立医院综合改革取得新成效，继续优化医疗卫生资源布局，推进分级诊疗体系和医疗联合体建设，提供全方位、全周期的卫生健康服务。实施公共体育“31166”建设工程，健全五级公共体育设施网络，促进体育设施结构均衡、城乡均质。加快军民深度融合发展，提高双拥共建水平。

3.积极创新社会治理。加强精神文明建设，健全文明城市长效管理机制，扩大道德讲堂影响力，不断提升市民文明素质和城市文明程度。启动全国和谐社区建设示范城创建，完善社区服务功能，培育各类社会组织，推进社区多元共治。加强和改进信访工作，完善社会矛盾纠纷有效预防和多元化解机制。健全立体化、信息化社会治安防控体系，注重完善联动融合、开放共治的社会治理模式，建设更高水平“平安常州”。严格落实安全生产责任制，加强食品药品环境安全监管，强化灾害监测预警和风险防控，严守金融稳定底线，坚决遏制重特大安全事故和群体性事件发生，确保社会和谐稳定。

支持工会、共青团、妇联等人民团体工作，加强外事侨务、对台事务、档案方志、民族宗教、防灾减灾、应急管理、气象、残疾人事业、老龄和关心下一代等工作。

与此同时，今年我们将办好以“三优三安两提升”（优质教育、优质医疗、优质养老、安心食品药品、安心饮用水、安心社会治安、提升生态绿城建设水平、提升大气环境质量）为重点的民生实事，让广大市民更好地享受改革发展成果。三、切实加强政府自身建设二号首长

新常态要有新状态，新时期要有新作为。我们将以更高的标准、更严的要求加快转变政府职能，努力构建符合法治精神、充满生机活力、更加廉洁高效的政务生态系统。

（一）坚持高效便民，着力建设服务型政府。深化简政放权、放管结合、优化服务改革，进一步推进行政审批“三集中三到位”，提高行政审批效率。稳妥推进相对集中许可权改革，优化建设项目“五联合一简化”联审联办机制，清理规范涉审中介、强制性检验检测，创造良好投资环境。打造全市政务服务“一张网”，推动政务服务事项“应上尽上、全程在线”。加快“一办四中心”建设，形成一站式的政务服务模式。加大政务公开力度，推进决策、执行、管理、服务、结果公开，不断完善“三合一”阳光政务平台，增强政务公开的影响力和舆论引导力。

（二）坚持依法行政，着力建设法治政府。把依法行政贯穿于政府工作的各个层面、各个环节，强化政府立法，善于利用法律手段管理经济社会事务。完善依法决策机制，充分发挥法律顾问在依法行政中的作用，健全重大行政决策公众参与和风险评估机制，确保政府决策科学化、法治化、民主化。深化综合行政执法体制改革，促进行政权力和执法力量下放，切实规范行政执法行为。强化行政权力监督，自觉接受人大依法监督、政协民主监督、司法监督、社会监督和舆论监督，加强审计监督，让行政权力在法治轨道和阳光下运行。

（三）坚持求真务实，着力建设创新型政府。把创新摆在政府工作的重要位置，以创新的理念谋划发展、创新的办法破解难题、创新的措施推动工作。创新公共服务供给，更多运用市场化、社会化方式，提升公共服务效率。创新政府服务方式，加强电子政务建设，充分发挥“政企通”作用，运用互联网、大数据，提高政府服务效能。把开拓创新和求真务实紧密结合起来，发扬“钉钉子”精神，一张蓝图绘到底，脚踏实地、苦干实干、善作善成，努力创造出经得起实践、群众和历史检验的工作业绩。大力弘扬“马上就办、真抓实干”的工作作风，狠抓执行落实，强化使命担当，健全激励和容错纠错机制，提升政府执行力和公信力。

（四）坚持从严治政，着力建设廉洁政府。把纪律和规矩挺在前面，严格落实政府系统党风廉政建设主体责任，坚持有腐必反、有贪必肃，以零容忍的态度坚决惩治腐败。加强公务员反腐倡廉教育，健全权力运行监督制约机制，努力形成不敢腐、不能腐、不想腐的体制机制。严格执行中央“八项规定”、省委“十项规定”和市委“30条意见”，坚决防止“四风”问题反弹。健全公共资源交易、政府投资管理、国有资本运作等制度体系，扎紧制度“笼子”，规范权力运行。切实厉行勤俭节约，严控“三公”经费支出，把更多财力用在急需的民生事业上。

各位代表，时代赋予我们重任，人民寄予我们厚望，唯有实干才能铸就新的辉煌。让我们紧密团结在以习近平同志为核心的党中央周围，在省委、省政府和市委的坚强领导下，解放思想、脚踏实地、真抓实干，为建设“强富美高”新常州而不懈奋斗！
